# Long Distance Dispersal of Zooplankton Endemic to Isolated Mountaintops - an Example of an Ecological Process Operating on an Evolutionary Time Scale

**DOI:** 10.1371/journal.pone.0026730

**Published:** 2011-11-10

**Authors:** Bram Vanschoenwinkel, Joachim Mergeay, Tom Pinceel, Aline Waterkeyn, Hanne Vandewaerde, Maitland Seaman, Luc Brendonck

**Affiliations:** 1 Laboratory of Aquatic Ecology and Evolutionary Biology, Katholieke Universiteit Leuven, Leuven, Belgium; 2 Research Institute for Nature and Forest, Geraardsbergen, Belgium; 3 Centre for Environmental Management, University of the Free State, Bloemfontein, South Africa; American University in Cairo, Egypt

## Abstract

Recent findings suggest a convergence of time scales between ecological and evolutionary processes which is usually explained in terms of rapid micro evolution resulting in evolution on ecological time scales. A similar convergence, however, can also emerge when slow ecological processes take place on evolutionary time scales. A good example of such a slow ecological process is the colonization of remote aquatic habitats by passively dispersed zooplankton. Using variation at the protein coding mitochondrial *COI* gene, we investigated the balance between mutation and migration as drivers of genetic diversity in two *Branchipodopsis* fairy shrimp species (Crustacea, Anostraca) endemic to remote temporary rock pool clusters at the summit of isolated mountaintops in central South Africa. We showed that both species colonized the region almost simultaneously c. 0.8 My ago, but exhibit contrasting patterns of regional genetic diversity and demographic history. The haplotype network of the common *B.* cf. *wolfi* showed clear evidence of 11 long distance dispersal events (up to 140 km) with five haplotypes that are shared among distant inselbergs, as well as some more spatially isolated derivates. Similar patterns were not observed for *B. drakensbergensis* presumably since this rarer species experienced a genetic bottleneck. We conclude that the observed genetic patterns reflect rare historic colonization events rather than frequent ongoing gene flow. Moreover, the high regional haplotype diversity combined with a high degree of haplotype endemicity indicates that evolutionary- (mutation) and ecological (migration) processes in this system operate on similar time scales.

## Introduction

Inselbergs are isolated rocky outcrops rising abruptly from the landscape. Often occuring as monoliths, they are characterized by contrasting and often more extreme environmental conditions compared to the surrounding plains [1], [2]. Rich in specific microhabitats, inselbergs provide niches for species that may be absent, rare or endangered in the surrounding habitat matrix [3], and the combination of spatial isolation and specific environmental conditions is known to promote endemism [4]. Besides harboring a peculiar terrestrial fauna and flora [5], the formation of eroded depressions near the summit of these mountains often gives rise to unique aquatic ecosystems that intermittently hold water after rains [6]. Generally occurring as clusters, these archipelagos of temporary rock pools situated on isolated mountaintops form a double insular habitat structured at three spatial scales with pools grouped in clusters, one or more pool clusters present on each inselberg and different inselbergs embedded in a matrix of inhospitable dryland [7].

In Southern Africa, fairy shrimp of the genus *Branchipodopsis* Sars 1998 (Crustacea: Anostraca) are typical inhabitants of rock pool habitats on the top of inselbergs [8], [9]. In order to survive dry periods they rely on dormant eggs (encysted embryos): small drought resistant propagules, which also represent the main dispersing life stage [10]. Fairy shrimp heavily depend on vectors for dispersal [11]. While amphibians [12], water flow [13] and notably wind [14] have been identified as important dispersal vectors operating at local scales (tens up to hundreds of meters), little is known about the frequency of passive dispersal and the identity of responsible vectors at larger spatial scales [15]. Candidate vectors mediating long distance dispersal of fairy shrimp dormant eggs between distant pool clusters and inselbergs include birds [16] and wind [14], [17]. Two experimental studies suggest that aquatic insects can also transport dormant eggs of zooplankton such as water fleas in controlled experimental settings [18], [19] but the scale of this process as well as its importance in natural systems remain unclear. Due to the practical difficulties in obtaining dispersal data, particularly over long distances, dispersal dynamics of freshwater zooplankton are often inferred indirectly using molecular markers [20] or isotopes [21]. Genetic studies in different fairy shrimp species, for example, support the idea of frequent dispersal and gene flow over short distances such as within pool clusters [13], [22], [23], [24], [25]. In contrast, surprisingly little is known about the frequency of dispersal and gene flow among remote or isolated aquatic habitats such as mountain pools or groundwater aquifers and over large spatial scales [26], [27], [28]. A suitable approach to detect historic long distance dispersal (LDD) events is to study the geographic distribution of genetic lineages [29], [30].

Recent studies suggest a convergence of time scales between ecological and evolutionary processes which is usually explained in terms of rapid micro evolution resulting in evolution on ecological time scales [31], [32]. A similar convergence, however, can also occur when slow ecological processes such as rare LDD events take place at evolutionary time scales.

Here we use partial sequences of the mitochondrial protein coding cytochrome oxidase subunit 1 gene (*COI* or *cox1*) to investigate the frequency of LDD in two fairy shrimp: the locally common *Branchipodopsis* cf. *wolfi* Daday, 1910 and the rarer *Branchipodopsis drakensbergensis* Hamer and Appleton, 1996; both of which are endemic to remote rock pool habitats in central South Africa and often occur as mixed populations. Since both species are absent from aquatic habitats in the surrounding lowland areas, colonization and exchange of individuals between inselbergs, which are usually separated by tens of kilometers, implies LDD.

Although conclusive evidence is currently unavailable, preliminary investigations including morphological analyses and analyses of species distributions along main environmental gradients revealed no indications for environmental or temporal niche segregation [8], (Vandewaerde, unpub. data). Crossing experiments also indicate that the two species, which differ on average 26% at the *COI* gene, do not hydridize (Vanschoenwinkel unpub. data). *B.* cf. *wolfi* represents a taxon which is morphologically very similar to the *B. wolfi* a widespread species in Southern Africa [8]. Preliminary phylogenetic analyses, however, indicate that the former differs on average 14% at the *COI* gene from confirmed *B. wolfi* populations from Botswana (Genbank Accession No. GU171358). Based on accepted molecular thresholds indicative of species level differentiation in fairy shrimp at the *COI* gene (10–6%) this taxon should probably be considered a separate species awaiting formal description [33], [34]. Previous research has shown that within pool clusters both fairy shrimp species are dispersed by amphibians (*Xenopus laevis*), water flow through eroded channels and wind [22]. Although observations are very rare, some bird species such as ibises and small song birds are known to visit rock pools in the area (Vanschoenwinkel, pers. obs.) and could potentially transport fairy shrimp resting eggs at regional scales.

Based on the current distribution of genealogical lineages, topology of haplotype networks, partitioning of genetic variation and isolation by distance analyses, we discuss the demographic history of the species in the area and the frequency of dispersal at different spatial scales. Finally, to assess how the time scale of a slow ecological process such as LDD measures up to the time scale of an evolutionary process such as mutation, we compare mutation - and migration rates.

We specifically test the following hypotheses:

1. Since previous work demonstrated frequent passive dispersal and gene flow within pool clusters [14], [22], we hypothesize that phylogeographic signals and haplotype endemicity in this spatially hierarchical habitat will be mainly restricted to higher levels of spatial organization such as pool clusters and inselbergs rather than individual populations.
2. We anticipate similar rates of LDD for both species, given that the eggs are morphologically similar and are likely to share the same dispersal vectors.
3. Since avian vectors are scarce in this area, if wind dispersal is important, LDD events could be constrained by the dominant wind direction.
4. Due to the isolated nature of inselberg habitats we expect that migration rates among inselbergs will be very low, possibly of the same order of magnitude as mutation rates in the studied gene, illustrating a convergence of the time scales on which these two main drivers of (neutral) genetic diversity operate.

## Materials and Methods

### Ethics statement

All samples were collected on private land with explicit permission of the respective landowners. Collection and export permits were granted by the Free State Province Department of Tourism, Environmental and Economic affairs: permit no.: HK/P1/07375/001. Collected animals were anaesthetized in carbonized water before transfer to ethanol.

### Study area

**Figure 1:**
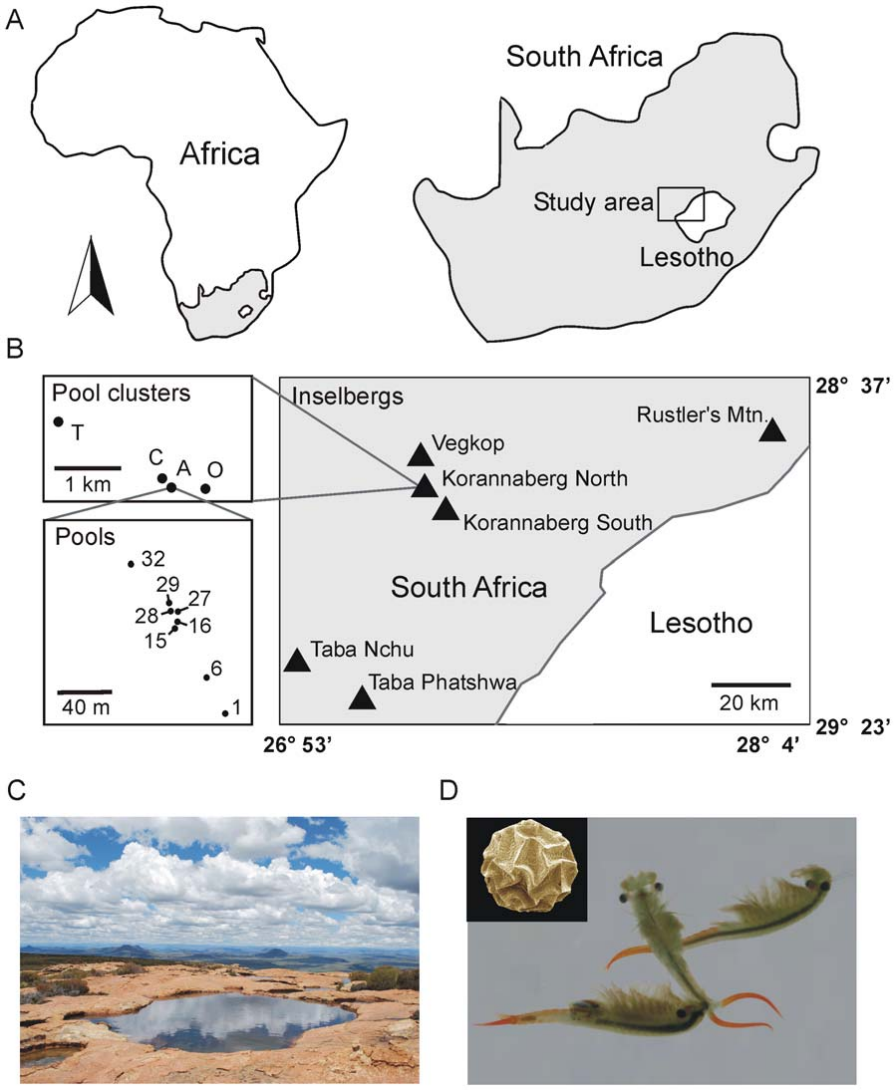
Study area and model organism. (a) Location of the study area in Africa. (b) Map of the study area illustrating the spatial hierarchical structure of the habitat: location of different inselbergs housing pool clusters with fairy shrimp populations in the regional landscape (right), location of different pool clusters on a single inselberg (Korannaberg North) (top left) and different populations embedded in one pool cluster (cluster A, Korannaberg North) (bottom left). (c) Typical rock pool located at the summit of the inselberg Taba Phatshwa, Free State, South Africa (Photo courtesy of Bram Vanschoenwinkel), (d) *Branchipodopsis* fairy shrimp (Photo courtesy of Dirk Ercken); right: B. cf. *wolfi* male, middle: *B. drakensbergensis* male, left: B. sp. female. inset: scanning electron microscopic picture of a *B.* sp. dormant egg (230 µm diameter). Females and dormant eggs of both species cannot be distinguished morphologically.

The landscape in the east of South Africa's Free State province is well known for the presence of inselbergs rising up from the wide open grasslands (Fig. 1a, b). These sandstone mountains are considered isolated satellites of the Drakensberg mountain range [33] but differ from the former in that they are not basalt capped. The youngest sandstone formation exposed at the summit (Clarens formation) tends to form calcareous concretions from trapped organic matter [35]. Once exposed, these concretions weather faster than the surrounding sandstone, leaving depressions with near vertical sides which after rains are transformed into temporary aquatic habitats (Fig. 1c). Sandstone inselbergs are almost exclusively confined to an area north of the Drakensberg and the kingdom of Lesotho ranging from Thaba Nchu in the west as far as Rustler's Mountain in the East. All 35 inselbergs in this area with exposed sandstone rock slabs at the top were visited during this study in search of fairy shrimp populations. Both active communities in inundated pools and pool sediment were checked for the presence of fairy shrimp or their dormant eggs. Fairy shrimp distributions were limited to six inselbergs (Korannaberg North, KN; Korannaberg South, KS; Rustler's Mountain, RUS; Vegkop, VEG, Taba Nchu, TN and Taba Phatshwa, TP; Table 1, Fig. 1b). Other inselbergs lacked eroded depressions altogether or contained rock pool basins which were too shallow and, as a result, too ephemeral to allow for the establishment of fairy shrimp populations. As shown in previous research, shallow pools (basin depth <10 cm) presumably cannot hold water long enough for fairy shrimp to reproduce [36]. All rock pools occured in the same sandstone formation, which recurs at the summit of each mountain. Therefore, assuming that regional weathering and erosion rates are relatively constant, deeper pools are most likely older than shallower pools [37]. By far the largest sandstone formation in the region is the Korannaberg, situated between the towns of Excelsior and Marquard and consisting of two separate plateaus: a northwestern (7.6 km^2^) and a southeastern (31.7 km^2^) which can be considered separate inselbergs. While most inselbergs in the region only house a small number of pools near the summit (0–10), the northwestern plateau of the Korannaberg (Korannaberg North) is the only inselberg that boasts several spatially isolated clusters of up to 36 pools. Despite the much larger area, rock pools are very rare on the southeastern plateau (Korannaberg South) probably due to the scarcity of large exposed sandstone rock ledges.

**Table 1:** Overview of sample locations.

							Species present	
Inselberg	Code	Cluster	Latitude (S)	Longitude (E)	Cluster size (#pools)	# pools sampled	*B. drak.*	*B.*cf.*wolfi*
Rustler's Mtn.	RUS	RUS	28°43′26.4″	28°01′37.1″	2	2	0	1
Vegkop	VEG	VEG	28°47′34.1″	27°13′51.3″	8	2	0	1
Taba Nchu	TB	TB	29°15′13.2″	26°54′30.7″	3	2	0	1
Taba Phatshwa	TN	TP	29°20′02″	27°05′13.2″	5	2	0	1
Korannaberg South	KS	K	28°52′34.6″	27°17′19.4″	4	2	1	1
Korannaberg North	KN	C	28°51′09.5″	27°13′45.4″	10	2	1	1
		O	28°51′12.63″	27°14′4.3″	2	2	0	1
		T	28°50°39.4″	27°12′55.4″	10	2	1	1
		A	28°51′11.96″	27°13′48.59″	36	8	1	1

### Sample collection and processing

Fairy shrimp resting eggs were collected during a field survey that took place from July 27^th^ until August 14^th^ 2008 in the dry season. Populations were sampled at three spatially hierarchical scales: (a) populations on different inselbergs (b) populations from different pool clusters on the same inselberg and (c) populations within a single pool cluster.

In order to investigate genetic variation among inselbergs, two populations were sampled on each inselberg. To investigate variation among pool clusters, four pool clusters present on Korannaberg North were used since this is the only inselberg that harbors multiple pool clusters. Here, again, two populations were sampled in each cluster. Finally, to assess within-cluster variation, nine populations were sampled in one specific cluster (cluster A on Korannaberg North). This pool cluster is the largest in the region containing 36 large pool basins and comprises the deepest and presumably oldest pools [35]. An overview of the different populations sampled is provided in Table 1.

Dry sediment containing resting eggs was collected from pool basins using a shovel and a fine brush and transferred to plastic ziplock bags. *Branchipodopsis* resting eggs were isolated from the sediment under a stereomicroscope and stored in 100% ethanol. Genomic DNA was extracted for multiple individuals from each population making use of the HotSHOT protocol [38] for resting eggs. Only for populations from Cluster A on Korannaberg North adult specimens preserved on absolute ethanol were available from a previous sampling campaign in 2005. Nucleospin extraction kits (Macherey – Nagel) were used to extract DNA from these individuals.

### Genetic markers

A fragment of the protein-coding mitochondrial cytochrome *c* oxidase subunit I (*COI* gene) was sequenced for up to 16 individuals per population using polymerase chain reaction (PCR). However, due to occasional low densities of viable eggs in pool sediment and failed PCR reactions the number of individuals included in our analyses was lower for some populations (Table 2). PCR cycle settings were modified from [39]: 35 cycles of 3 min at 94°C (denaturation), 30 s at 50°C (annealing) and 90 s at 72°C (extension) followed by 1 cycle of 6 min at 72°C. Best results were obtained with MgCl_2_ concentrations of 2 mM. As initial amplification and sequencing was unsuccessful for a number of populations we developed sets of internal primers using the web application PRIMER3 [40] based on preliminary sequence data obtained from universal Metozoa primers [41]. The forward primer BranCOIF1: (5′-TGC CCA TGC GTT TGT TAT G-3′) and reverse primer BranCOIR1: (5′-AATCAGAAGAGGTGTTGATAGAGG-3′) proved to be most effective amplifying a fragment of 485 bp (excluding primers). Sequences were obtained making use of the ABI Prism Big Dye Terminator sequencing protocol and the ABI Prism BigDye Terminator Cycle Sequencing Reaction Kit (PE Applied Biosystems). PCR fragments were sequenced with the above-mentioned forward and reverse primers. Sequences were aligned and trimmed in BioEDIT [42]. All sequences were deposited in GenBank (Accession No. GU139707-139737 and GU13171358).

**Table 2:** Distribution of COI mtDNA haplotype for *B. drakensbergensis* and *B.* cf. *wolfi* among different pools, pool clusters and inselbergs.

			*B. drakensbergensis*											*B.* cf. *wolfi*																					
Inselberg	Clust.	Pop.	1	2	3	4	5	6	7	8	9	10	n	1	2	3	4	5	6	7	8	9	10	11	12	13	14	15	16	17	18	19	20	21	n
RUS	RUS	RUS1											0									14								1					15
VEG	VEG	VEG1											0		4			1														1			6
		VEG2											0	1	14			1																	16
TB	TB	TB1											0																	15					15
		TB2											0							1										7	1	3			12
TP	TP	TP1											0		2	5								5			1	2							15
		TP2											0		5			1	1				1	1				6							15
KS	K	K1				7							7												1	3									4
KN	C	C8	1	2									3		4		1															11			16
KN	O	O1											0		16																				16
KN	T	T1							1				1																					7	7
		T2					3	1	2	1	2	1	10																					4	4
KN	A	1											0		14																				14
		6	4	3	6								13		1																				1
		15											0		3						1								1			7	1		13
		16											0		4						2								2			6			14
		27											0		5						3											6			14
		28	11	2									12		7						2														9
		29											0		7						2	1										6			16
		32											0		5		1															4			10

Haplotypes recovered for each species are numbered.

### Data analysis

In order to remove anomalies and export consensus sequences based on the two complementary electropherograms obtained for each individual, SeqScape© software v. 2.5.0 (Applied Biosystems) was used. Haplotype networks for each of the two species were created in Network 4.0 [43]. In order to test whether the topology of the network was robust this analysis was repeated using the TCS software package [44]. Networks were constructed using a maximum parsimony criterion (statistical parsimony network).

In order to visualize the degree of haplotype endemicity and haplotype sharing among sites we used pie charts to plot the distribution of different haplotypes on a map of the study area. Both at the level of pools, pool clusters and inselbergs, haplotype endemicity was calculated as the percentage of haplotypes unique to that specific locality.

Given the fact that dispersal rates are low enough, information about the route and the direction of historic long distance dispersal events can be derived from two sources: network topology and patterns of haplotype sharing. In star-shaped networks with one clear central haplotype linked to a specific locality, this can be considered the source of the initial colonization. In contrast, in the case of haplotype sharing, which reflects more recent migrations, directionality can be deduced when haplotypes in the source habitat are more closely related to the shared haplotype than the destination. If this difference was not clear, directionality could not be determined.

Historical demographic structure of both species was studied using Fu's F tests for deviations from mutation-drift equilibrium [45] in Arlequin 3.5.1.2 [46], using 10 000 permutations. For neutrally evolving sequences they suggest sudden demographic expansions when significantly negative, or contractions when significantly positive. Using a molecular clock of 1.4 to 2.6% per My [47], we estimated the timing of the diversification among the most distant haplotypes, and the average age between all haplotypes within species. The average age was estimated from the parameter Tau of the mismatch distribution [48] obtained in Arlequin 3.5.1.2. Given that *B. cf wolfi* seems entirely restricted to the sampled range, and that it is unlikely that *B. drakensbergensis* has colonized the Korannaberg multiple times from independent sources (the nearest known occurrence is more than 400 km away; [49]) we can assume that all diversification occurred within the sampled region.

Analysis of molecular variance (AMOVA) [50] was performed in Arlequin 3.5.1.2 to assess the partitioning of variation in haplotype diversity at the three levels of spatial organisation: individual populations, pool clusters and inselbergs. Haplotype richness was calculated as the number of haplotypes per population. Estimates of haplotype diversity (*h*) and nucleotide diversity (π) were calculated in Arlequin 3.5.1.2 for each pool, each pool cluster and each inselberg. Since dominant winds consistently blow from the north and to a lesser extent from the northwest [14], we hypothesized that genetic diversity measured in different pool clusters on different mountains could be positively correlated with latitude (which in the southern hemisphere increases in southerly direction). Possible relations between habitat characteristics and different genetic diversity estimates were explored using general linear models constructed in R (R Development Core Team, 2011; http://R-project.org). Independent variables in these analyses included altitude (m above sea level), longitude and latitude (decimal degrees), habitat size (pool surface area in m^2^), habitat duration (basin depth in cm). In rock pools habitat duration is primarily determined by depth of the pool basin hence we can use this variable as a proxy [51]. This analysis was performed both at the level of populations, pool clusters and inselbergs. For analyses at higher levels of spatial organization (pool clusters, inselbergs) both extreme (min, max) and average values of pool attributes (basin depth, surface area) in each pool cluster or inselberg were used.

For analyses using inselbergs as units, the frequencies of haplotypes found on each inselberg were used to calculate indices. A similar procedure was followed for analyses using pool clusters unites. Analyses using pool clusters as independent units were only performed for Korannaberg North since this is the only inselberg which harbors multiple pool clusters. Analyses at the level of individual populations were performed for a reduced dataset containing only the populations in the intensively sampled pool cluster A on Korannaberg North.

Isolation by distance (IBD) analyzes were performed using the web application IBDW [52] specifying Slatkin's *M* [53] as a measure of genetic distance. This application performs Reduced Major Axis (RMA) regression analysis and Mantel tests [54]. Again, this analysis was carried out at three different spatial scales testing for the presence of IBD among pools in one pool cluster (cluster A on Korannaberg North), between pool clusters on a single inselberg (Korannaberg North) and among different inselbergs.

From the haplotype networks, it is possible to count both the total number of mutation events (Mu) as well as the number of haplotypes (HR) that originated through these mutation events. Haplotype networks, however, also typically contain missing haplotypes. Assuming that new haplotypes are generated solely in a single step fashion, these virtual haplotypes represent those that disappeared in the course of time as a result of genetic drift. As a result, comparing the total number of mutation events (Mu) to the number of haplotypes (HR) provides a means to assess the relative role of genetic drift and the ratio HR/Mu can be used as a measure of the effect of genetic drift on the total remaining diversity since the onset of diversification.

Additionally, for each species, the total number of migration events (m_r_) can be assessed by counting the number of haplotypes shared among inselbergs added to the number of haplotypes derived from haplotypes that occur on other inselbergs. Due to genetic drift, however, an unknown number of historical migration events between inselbergs remain unnoticed. Yet, because genetic drift has the same effect on genetic diversity irrespective of how the diversity originated (mutation or migration), the number of total expected migration events (m_e_) in the course of history calculated, based on the number of detected migrations corrected for those lost by drift using the equation m_e_ = m_r_ × (Mu/Hr). This value of m_e_ can then be used to estimate the rate of migration over time, if the start of the genetic diversification can be dated using a molecular clock.

## Results

After trimming, a 485 bp alignment was obtained. The alignment was unambiguous and did not contain indels. A total of 31 different *COI* mtDNA haplotypes was detected: 21 belonging to *B.* cf. *wolfi* and 10 to *B. drakensbergensis*. Their distribution among different pools, pool clusters and inselbergs is provided in Table 2.

**Figure 2:**
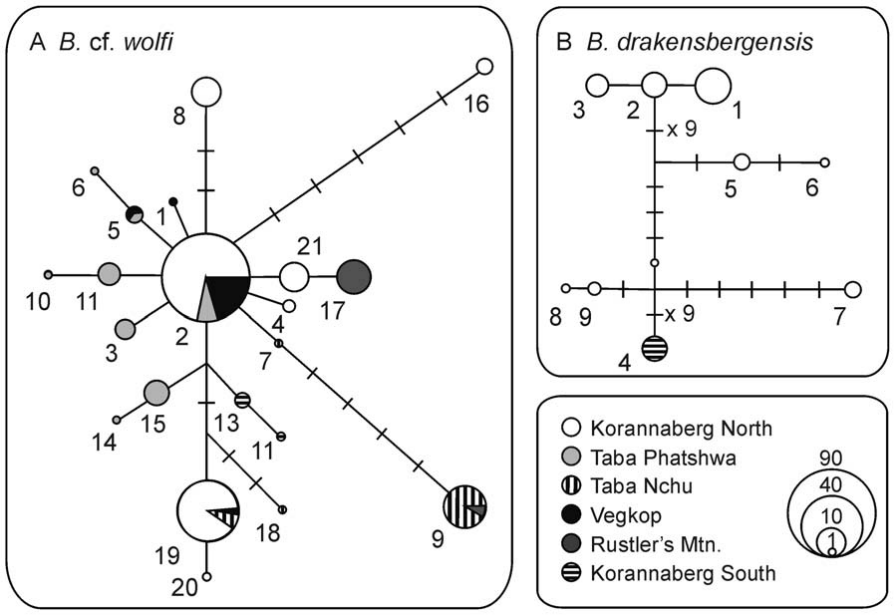
Haplotype networks. Statistical parsimony networks showing the evolutionary relations and geographic occurrence of (a) *Branchipodopsis* cf. *wolfi* and (b) *Branchipodopsis drakensbergensis* mitochondrial haplotypes numbered according to Table 1. Pie charts illustrate distribution and relative abundance of each haplotype on different inselbergs. Size of circles is proportional to the number of individuals included (bottom right). Perpendicular black dashes represent hypothetical intermediate haplotypes. The numbers of such dashes between two haplotypes indicates the number of mutational steps involved.

**Figure 3:**
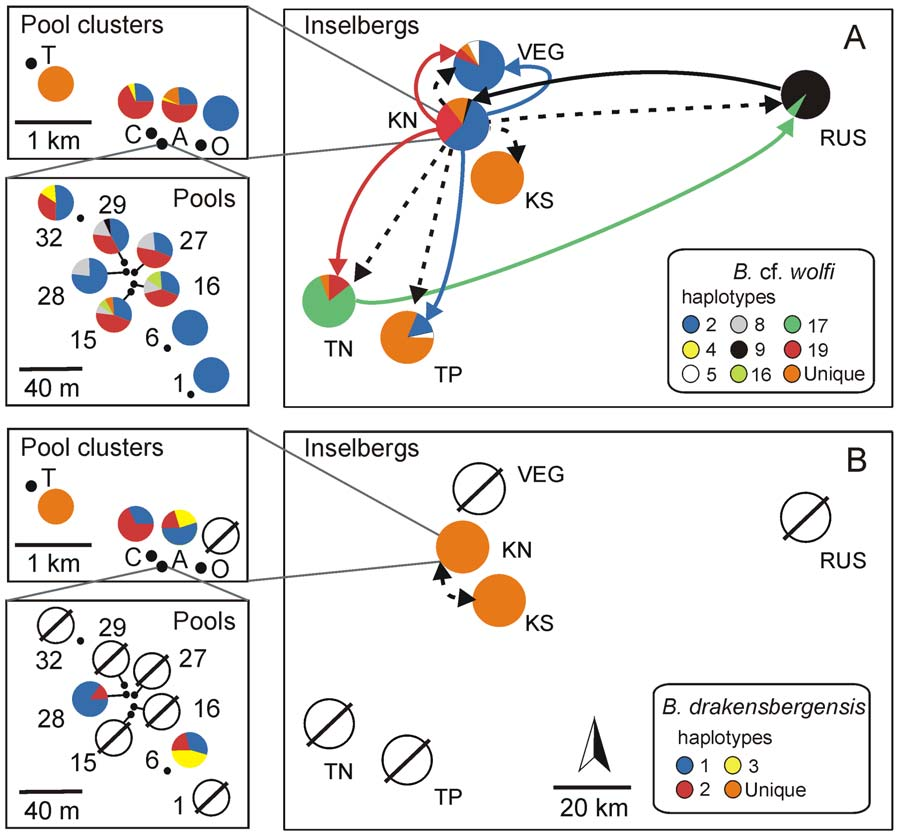
Haplotype distributions indicating long distance dispersal events. Geographic distribution of haplotypes of (a) *Branchipodopsis* cf. *wolfi* and (b) *Branchipodopsis drakensbergensis* among inselbergs (right), among the different pool clusters on Korannaberg North (top left) and among different pools within pool cluster A on Korannaberg North (bottom left). Pie charts depict relative abundance of haplotypes at each site. The proportion of haplotypes unique to a certain locality is highlighted in orange. A Ø symbol indicates that the respective species was not found at this locality. Arrows indicate long distance dispersal events detected in our analyses. Dashed arrows mark initial colonization events based on the topology of the haplotype network, colored arrows illustrate more recent migration evidenced by haplotype sharing. Direction of dispersal could not be determined for haplotypes 5, thus no arrow was plotted.

Haplotype networks were built to elucidate the genetic structure within each species (Fig. 2). The network of *B.* cf. *wolfi* (Fig. 2a) consisted of 21 haplotypes and was more or less star-shaped with the most abundant haplotype (h2) placed in the centre. This haplotype was found on three of the six inselbergs sampled: Korannaberg north, Thaba Phatshwa and Vegkop. In contrast, the haplotype network of *B. drakensbergensis* (Fig. 2b) did not depict starlike patterns or central haplotypes but rather consisted of ten more distantly related haplotypes that were overall less abundant and restricted to three pool clusters on Korannaberg North and the only cluster present on Korannaberg South (Fig. 3b).

In *B.* cf. *wolfi* four haplotypes (19%; h2, h5, h9, h19) were shared among distant inselbergs indicative of long distance dispersal. Two (9.5%; h4, h19) were shared among two pool clusters while one (4.8%; h2) was shared among three pool clusters on Korannaberg North. Three abundant haplotypes were shared among respectively, eight (100%; h2), and five (16%; h8, h19) of the populations sampled in cluster A on Korannaberg North. 76% of the haplotypes were exclusive to specific inselbergs, 67% were specific to pool clusters while 52% were restricted to a single population (Fig. 3a; Table S1).

On the other hand in *B. drakensbergensis*, which is restricted to two inselbergs (KN and KS), no haplotypes were shared among inselbergs. Two haplotypes (20%; h1, h2) were shared among two pool clusters as well as among two populations in cluster A on Korannaberg North. 80% were exclusive to pool clusters while 70% of the haplotypes were population specific (Fig. 3b; Table S1).

The test of population expansion or contraction (Fu's Fs) was inconclusive for *B.* cf. *wolfi* as it showed no significant deviation from mutation-drift equilibrium (Fs = −2.85, Prob. _sim Fs < = obs. Fs_ = 0.22). For *B. drakensbergensis* Fu's Fs indicated a marginally significant demographic contraction (Fs = 5.15, Prob_sim_Fs>obs_Fs_ = 0.052). Using the most abundant haplotype (haplotype 2; Table 2) as root of the network, the start of the genetic diversification of *B.* cf. *wolfi* was estimated at 826 ky B.P. (1 187–634 ky B.P.), whereas the average age between all haplotypes was estimated at 308 ky (440–237 ky). For *B. drakensbergensis* a virtual root of the network was taken as half the distance between the two most distant haplotypes as we could not identify a clear root to the network. This dated the origin of the diversification within *B. drakensbergensis* at 876 ky B.P. (1 252–674 ky B.P.). The average age between all haplotypes was estimated at 800 ky (1 143–615 ky).

Due to the restricted geographic distribution (two inselbergs) of *B. drakensbergensis*, and limited number of populations (n = 6) resulting in limited statistical power, the remaining analyses are only presented for *B.* cf. *wolfi*. The AMOVA results indicated that the genetic variation was dominantly structured among inselbergs (43%) and, unexpectedly, among populations (42%) while 15.2% of the remaining variation was distributed among pool clusters. No significant relations between habitat characteristics and any of the calculated diversity indices (haplotype richness, *h*, π) could be confirmed at the level of individual inselbergs, pool clusters or populations within a pool cluster. No significant isolation by distance patterns were detected among pool clusters on the same inselberg (r = 0.15, P = 0.52) or among pools in the same pool cluster (r = −0.15, P = 0.3). Among inselbergs the correlation between geographic distance (LOG transformed) and genetic similarity could be considered marginally significant (r = −0.51, P = 0.08).

For *B.* cf. *wolfi* with a haplotype richness of 21, the number of mutational steps (Mu) in the network amounted to 36, resulting in a Hr/Mu ratio of 0.58 indicating that 58% of the genetic diversity was lost due to genetic drift. A total of 11 residual migration events was detected, yielding a total expected historical number of migration events of 19. Given that the onset of diversification was estimated at 826 ky B.P., this gives an average long-distance among inselbergs rate of migration of one individual every 43.5 ky.

For *B. drakensbergensis*, a haplotype richness of 10 and 39 mutational steps, results in a Hr/Mu ratio of 0.256. A single residual migration event among inselbergs was detected, yielding an estimated grand total of one migrant every 225 ky, on average. Mutation rates for the sequenced gene fragment according to molecular clocks, in turn, range from 0.7 to 1.3 mutations per ky or one mutation every 80–150 ky.

## Discussion

In this study, the genetic structure of two fairy shrimp species endemic to isolated rock pool clusters at the top of inselbergs in central South Africa was investigated. The rare *B. drakensbergensis*, was represented by a small number of distantly related lineages surviving in relict populations in a restricted range. The common *B.* cf. *wolfi*, on the other hand, exhibited higher haplotype diversity, a more stable demographic history and a wider geographic distribution.

### Spatial structuring of genetic variation

Current results based on mitochondrial sequence data suggest that, for rock pool inhabiting fairy shrimp, most genetic variation seems to be partitioned at the regional scale between inselbergs and to a lesser extent between different pool clusters. To some extent, this is consistent with conclusions of earlier studies describing allozymic variation [13], [25]. In *Branchinecta coloradensis*, which inhabits pools in the Rocky Mountains (USA), for instance, high levels of allozymic differentiation were observed between populations in different valleys (5–10 km apart) while on local scales (0–100 m) populations were genetically similar [23]. Similar patterns of low levels of allozymic differentiation in pool clusters and higher differentiation among pool clusters were observed in the African fairy shrimp *Branchipodopsis wolfi* [13], [24], [25]. Unexpectedly, however, a substantial amount of genetic variation in our dataset including unique haplotypes was detected at the population level, suggesting that genetic homogenization within pool clusters does not seem to occur even though previous studies measured high passive dispersal rates of fairy shrimps within rock pool clusters facilitated by wind and water flow [14], [22]. This could thus be an example of the well known dispersal-gene flow paradox in freshwater organisms [55], which emphasizes that both priority effects and biotic barriers, potentially reinforced by local adaptation, may drastically reduce establishment success of incoming dispersers and lead to low levels of gene flow in the presence of high dispersal rates [29]. Low variation among pool clusters suggested by the AMOVA results most likely results from the fact that multiple pool clusters were only present on one of the studied inselbergs.

The high levels of genetic diversity and the presence of many unique haplotypes linked to specific localities, found on the limited geographic scale of this study system is remarkable when compared to other species of large branchiopods in temperate and Mediterranean regions [30], [56], [57]. Reasons for this discrepancy probably lie in the fact that subtropical species have been less affected by random genetic processes that are typically associated with glaciations (genetic drift, bottlenecks, founder events) than their counterparts in more temperate regions [58], the fact that Southern Africa has been a relatively stable region throughout much of its recent history [59] and the isolated nature of inselbergs promoting endemism [4].

### Reconstructing historic long distance dispersal

In *B.* cf. *wolfi*, the abundance of the two most common haplotypes (haplotypes 2 and 19; Table 2) on Korannaberg North and their central position in the gene genealogy indicate that the origin of the radiation of *B.* cf. *wolfi* in the region is most likely situated on this inselberg. This is not surprising as the pool basins on this mountain are the deepest and therefore probably also the oldest [37] in the region. From this inselberg the species colonized five other inselbergs implying at least five effective long distance dispersal events over distances ranging from 6 up to 124 km. Additionally, five haplotypes are shared among inselbergs resulting coexistence of remotely related haplotypes notably on Thaba Nchu and Rustler's mountain as well as complex relations between haplotypes on Korannaberg North and Thaba Phatshwa which are testimony of reciprocal colonization events and secondary contact between populations (Fig. 3a). In contrast, the origin of the radiation in the rare *B. drakensbergensis* could not be pinpointed (Fig. 3b). Currently the species is confined to two inselbergs separated by 6 km. Since no haplotypes are shared among inselbergs this distribution reflects only a single long distance migration event. The reason why, contrary to our expectations, long distance dispersal was much less common in *B. drakensbergensis* than in *B.* cf. *wolfi* is probably related to the fact that the former experienced a genetic bottleneck, and currently consists of fewer surviving populations resulting in a lower probability to detect long distance dispersal events.

Although the diversification in both species in the area started nearly simultaneously around 800 ky B.P., both the topology of the haplotype networks as well as tests of population expansion and contraction suggest that the species have a different demographic history. The demographic history of *B.* cf. *wolfi* can be considered stable (no significant deviation from mutation-drift equilibrium), whereas *B. drakensbergensis* most likely experienced a demographic bottleneck. Although we cannot provide conclusive answers at this point, differences in niche requirements, dispersal capacity, or a competitive disadvantage could explain why, from the two species, only *B. drakensbergensis* experienced a bottleneck and why this species is currently only found on a subset of the inselbergs and in a subset of the rock pools inhabited by *B.* cf. *wolfi.* Differences in dispersal capacity, however, do not provide a satisfactory explanation since the dormant eggs, which represent the dispersing life stages, are apparently identical in both species [60]. What is more, based on their ecology, morphology and phenology there are no obvious indications for niche differentiation [8].

### Do dominant winds constrain long distance dispersal?

Being small and inconspicuous rock pool habitats are not very attractive to bird life, compared to larger wetlands and lakes. With exception of some small song birds and a single observation of a visiting ibis (*Bostrychia hagedash*) no other birds have been observed visiting rock pools in the area. On the other hand, the area is characterized by very strong winds which were shown to facilitate short distance dispersal within pool clusters, we considered the possibility of long distance dispersal mediated by wind [14], [22]. A potential importance of wind mediating long distance dispersal could not be confirmed. Haplotype networks reflected that gene flow was not constrained by the dominant wind direction with evidence of LDD detected in all wind directions. Genetic diversity also did not accumulate downwind in the South of the study area. Such results, however, are expected for populations that are in mutation-drift equilibrium and where migration is less important than mutation (such as for *B.* cf. *wolfi*). Genetic diversity is therefore more a function of population size and the associated rate of genetic drift, rather than of the rate of immigration. This result, however, does not rule out that some of the LDD events detected in this study could be mediated by wind, since these events are often associated with extreme events such as storms and whirlwinds rather than standard conditions [61]. Additionally, interpretations relying on recent observations only may not fully elucidate the colonization history of inselbergs by fairy shrimp. The current absence of water birds and rock pool visiting birds in general, for instance, does not rule out that sporadic LDD mediated by birds occured during the long history of the species in the area [15].

### Isolation by distance patterns

Additional information about the scale of dispersal comes from isolation by distance (IBD) analyses. No significant IBD could be demonstrated in this study. Only at the largest (between inselberg) scale a marginally significant trend points to the possible presence of IBD. Absence of IBD is usually explained in terms of frequent dispersal or strong founder effects [62] and both processes are likely to be important here. Again, high dispersal rates over short distances measured in earlier studies [13], [22] can explain absence of IBD at a local, within cluster, scale. At regional scales, on the other hand, haplotype networks illustrate that gene flow among inselbergs is extremely rare. Similar conclusions were drawn by Muñoz and coworkers [29] who reported IBD despite the fact that gene flow between *Artemia salina* populations along the Mediterranean sea was very limited. Consequently, IBD patterns were not explained in terms of actual gene flow but as the result of persistent founder effects retaining genetic differentiation: a pattern that may be common in many other passively dispersed freshwater invertebrates [56], [62].

### Balance between migration and mutation rates

Based on our estimates of the rate of effective dispersal over time, it appears that migration rates were of the same order of magnitude as mutation rates in *B.* cf. *wolfi*, and nearly five times smaller in *B. drakensbergensis*. These calculated migration rates are relatively coarse estimates, but at the very least they indicate that effective long distance migration events are extremely rare. Initially, we expected similar migration rates for both species, given that the eggs are likely to share dispersal vectors. The much lower estimates for *B. drakensbergensis* may be due to the fact that it underwent a demographic contraction and significantly departed from mutation-drift equilibrium, and because the species is so rare in the region. Our data thus suggest that occasional haplotype sharing results from very rare effective dispersal events of a stochastic nature. These findings are relevant in the light of the evolving metacommunities concept [63], [64], which suggests a convergence of time scales at which ecological and evolutionary processes occur. While other studies mainly focused on the importance of rapid micro-evolution and convergence of evolutionary time towards rapid ecological time [31], [32], we report a decrease of the rate of a typical ecological process such as dispersal towards the rate at which evolutionary processes such as mutation are typically perceived. Currently, the general view among researchers is that, compared to standing genetic variation and migration, mutation is too slow to be an important factor determining local genetic diversity as well as the response of populations to environmental change. [65], [66]. However, as shown in this study mutation can be important in isolated habitats where lack of gene flow allows for a lot of time for local adaptation based on variation generated by mutation [67].

## Supporting Information

Table S1 Overview of the number of haplotypes (No. H) and percentage of exclusive haplotypes (haplotype endemicity; HE) present at the level of inselbergs, pool clusters and populations.
(DOCX)
