# Supplementary material for: Long Distance Dispersal of Zooplankton Endemic to Isolated Mountaintops - an Example of an Ecological Process Operating on an Evolutionary Time Scale
Source: PLoS One. 2011 Nov 10;6(11):e26730. doi: 10.1371/journal.pone.0026730 (PMC3213101; doi:10.1371/journal.pone.0026730)
Supplement: Table S1 — Overview of the number of haplotypes (No. H) and percentage of exclusive haplotypes (haplotype endemicity; HE) present at the level of inselbergs, pool clusters and populations. (DOCX) [file pone.0026730.s001.docx]

**Online supporting information :** *Table S1. Overview of the number of haplotypes (No. H) and percentage of exclusive haplotypes (haplotype endemicity; HE) present at the level of inselbergs, pool clusters and populations.*

|  |  | Species | | |  |  |  | Species | | |  |  |  | Species | | |  |
| --- | --- | --- | --- | --- | --- | --- | --- | --- | --- | --- | --- | --- | --- | --- | --- | --- | --- |
|  | cf. *wolfi* | |  | *drakensbergensis* | |  | cf. *wolfi* | |  | *drakensbergensis* | |  | cf. *wolfi* | |  | *drakensbergensis* | |
| Inselberg | No. H | HE (%) |  | No. H | HE (%) | Cluster | No. H | HE (%) |  | No. H | HE (%) | Population | No. H | HE (%) |  | No. H | HE (%) |
| RUS | 2 | 0 |  | 0 | 0 | RUS | 2 | 0 |  | 0 | 0 | RUS1 | 2 | 0 |  | 0 | 0 |
| VEG | 4 | 25 |  | 0 | 0 | VEG | 4 | 25 |  | 0 | 0 | VEG1 | 3 | 0 |  | 0 | 0 |
| TB | 4 | 50 |  | 0 | 0 | TB | 4 | 50 |  | 0 | 0 | VEG2 | 3 | 33,3 |  | 0 | 0 |
| TP | 8 | 75 |  | 0 | 0 | TP | 8 | 75 |  | 0 | 0 | TB1 | 1 | 0 |  | 0 | 0 |
| KS | 2 | 100 |  | 1 | 100 | K | 2 | 100 |  | 1 | 100 | TB2 | 4 | 50 |  | 0 | 0 |
| KN | 8 | 50 |  | 9 | 100 | C | 3 | 0 |  | 2 | 0 | TP1 | 5 | 60 |  | 0 | 0 |
|  |  |  |  |  |  | O | 1 | 0 |  | 0 | 0 | TP2 | 6 | 33,3 |  | 0 | 0 |
|  |  |  |  |  |  | T | 1 | 100 |  | 6 | 100 | K1 | 2 | 100 |  | 1 | 100 |
|  |  |  |  |  |  | A | 7 | 42,9 |  | 3 | 66,6 | C8 | 3 | 0 |  | 2 | 0 |
|  |  |  |  |  |  |  |  |  |  |  |  | O1 | 1 | 0 |  | 0 | 0 |
|  |  |  |  |  |  |  |  |  |  |  |  | T1 | 1 | 0 |  | 1 | 0 |
|  |  |  |  |  |  |  |  |  |  |  |  | T2 | 1 | 0 |  | 6 | 83 |
|  |  |  |  |  |  |  |  |  |  |  |  | 1 | 1 | 0 |  | 0 | 0 |
|  |  |  |  |  |  |  |  |  |  |  |  | 6 | 1 | 0 |  | 3 | 33,3 |
|  |  |  |  |  |  |  |  |  |  |  |  | 15 | 5 | 20 |  | 0 | 0 |
|  |  |  |  |  |  |  |  |  |  |  |  | 16 | 4 | 0 |  | 0 | 0 |
|  |  |  |  |  |  |  |  |  |  |  |  | 27 | 3 | 0 |  | 0 | 0 |
|  |  |  |  |  |  |  |  |  |  |  |  | 28 | 2 | 0 |  | 2 | 0 |
|  |  |  |  |  |  |  |  |  |  |  |  | 29 | 4 | 0 |  | 0 | 0 |
|  |  |  |  |  |  |  |  |  |  |  |  | 32 | 1 | 0 |  | 0 | 0 |
| TOTAL |  | 76 |  |  | 100 |  |  | 66,6 |  |  | 80 |  |  | 52 |  |  | 70 |
|  |  |  |  |  |  |  |  |  |  |  |  |  |  |  |  |  |  |
